# Antibiotic Treatment Response of Chronic Lung Diseases of Adult Sheep in the United Kingdom Based upon Ultrasonographic Findings

**DOI:** 10.1155/2014/537501

**Published:** 2014-05-27

**Authors:** Phil Scott

**Affiliations:** Division of Veterinary Clinical Sciences, R(D)SVS, University of Edinburgh, Easter Bush, Roslin, Midlothian EH25 9RG, UK

## Abstract

Examination of the lungs of adult sheep with chronic respiratory diseases was readily achieved using both 5 MHz linear and sector scanners. Superficial lung abscesses in eight sheep appeared as anechoic areas containing multiple hyperechoic dots bordered distally by a broad hyperechoic capsule. Unilateral fibrinous pleurisy (2 sheep) appeared as an anechoic area containing a hyperechoic latticework. Ovine pulmonary adenocarcinoma (OPA) lesions appeared as sharply demarcated hypoechoic areas in the lung parenchyma initially in the cranioventral lung lobes (21 sheep) with lesions also present in the caudodorsal diaphragmatic lobe (11 sheep); abscesses and areas of calcification within the OPA tumour mass were also identified. Daily treatment with procaine penicillin for 30 consecutive days was successful in both sheep with unilateral fibrinous pleurisy and six sheep identified with superficial lung abscesses measuring 2–8 cm in diameter; only one of two sheep with more extensive lesions recovered. Auscultation of the chest failed to detect adventitious sounds in any of the ten sheep with lung abscesses; normal breath sounds were reduced over the area of fibrinous pleurisy; no pleuritic rubs were heard. Wheezes and crackles auscultated in some OPA cases and did not correlate well with lesions detected ultrasonographically.

## 1. Introduction

An aetiological approach to the diagnosis, treatment, and control of respiratory diseases affecting sheep is often adopted in review articles [1] and book chapters [2, 3] but such precise classification is unrealistic in most general veterinary practice situations because of cost and access to specialised laboratory facilities. Clinical signs vary with the stage of the respiratory disease process and are not pathognomonic for particular aetiological agents [4]. Terms such as “chronic pasteurellosis” are commonly used to describe respiratory disease associated with weight loss with the recommendation of oxytetracycline therapy but there is little clinical evidence for such a diagnosis. Furthermore, detailed gross necropsies undertaken on farm prove difficult to interpret especially when complicated by autolytic change; histopathological examination is not often undertaken for cost reasons.

Individuals with chronic respiratory diseases are in poorer body conditions and have a higher respiratory rate than other sheep in the group [5]. The accuracy by which auscultation of the chest, as part of the standard veterinary examination, can detect, localise, and differentiate lung pathology has been questioned following comparison of adventitious sounds auscultated over normal lung areas and lesions of OPA [6]. Moderate to severe coarse crackles detected in advanced cases of OPA were audible over a larger area than lesion distribution identified during ultrasound examination and confirmed later at necropsy [5].

Publication of auscultated sounds recorded over specific respiratory tract pathologies, defined during simultaneous ultrasonographic investigation, has allowed clinicians to assess the value of auscultation of the chest performed as part of the standard veterinary clinical examination of sheep [5, 7] and cattle [8]. Auscultation did not detect any abnormal sounds in sheep with lung abscesses; unilateral pyothorax and marked fibrinous pleurisy caused attenuation of sounds relative to the contralateral normal lung. No sounds resembling the description of pleural frictions rubs were heard in cases of marked fibrinous pleurisy [5].

While ultrasonographic examination of the chest in cattle and sheep has been routinely undertaken in some veterinary schools for many years [9], this adjunct to clinical examination also has great potential in farm animal practice because the examination takes only 5 minutes and does not involve any laboratory fees. Many chronic respiratory infections of ruminants are not accurately diagnosed, and such cattle and sheep do not receive the correct antibiotic treatment [10, 11].

This paper describes the antibiotic treatment response in sheep with chronic lung diseases where the diagnosis was based upon ultrasonographic examination of the lungs using 5 MHz linear and sector scanners; no account was taken of auscultation findings. Lung pathologies were confirmed in those animals that failed to respond to treatment and were euthanased for welfare reasons. Necropsy examinations allowed comparison between sonographic findings and pathological changes.

## 2. Materials and Methods

The thirty one sheep included in this study originated from the University of Edinburgh's first opinion ambulatory practice and cases were referred by local veterinary practices. This study covered a two-year period (October 2011–October 2013 inclusive). To mimic the time constraints faced by farm animal practitioners, ultrasonographic examination of both sides of the chest, including skin preparation, totalled no more than 5 minutes for each type of ultrasound scanner and often took less time. Ultrasonographic examination of the chest was undertaken using both 5.0 MHz sector and linear transducers connected to a real-time, B-mode ultrasound machine (Aloka and BCF Technology Miniscan).

A 5 cm wide strip of skin was shaved on both sides of the thorax extending in a vertical plane from the point of the elbow to the caudal edge of the scapula corresponding to the 6th or 7th intercostal spaces. The prepared skin overlying the chest wall can be freely moved 3 cm which allowed examination of the caudal aspect of the lung field. The skin was soaked with warm tap water then ultrasound gel liberally applied to the wet skin to ensure good contact. The transducer head was firmly held at 90° to the skin overlying the intercostal muscles of the 6th or 7th intercostal spaces and the thorax was examined in the longitudinal (vertical) plane. The relatively large linear probe head was held on the chest wall at a slight angle to represent the angle of the intercostal spaces. It was important to visualise the echogenic (white) line of the normal visceral pleura at the most dorsal margin of the lung field before scanning the ventral areas of the chest. The visceral pleura was followed down the chest wall to identify the junction between normal lung and pathology where present.

The diagnosis of respiratory disease was confirmed at gross necropsy in 22 sheep. Bacteriology examinations were not undertaken because of prior antibiotic therapy. Ultrasonographic examination of 31 adult sheep with respiratory diseases associated with weight loss included OPA (21), fibrinous pleurisy (2 cases), and superficial lung abscesses (8 cases).

## 3. Results

### 3.1. Interpretation of Ultrasonographic Findings

The sonograms are presented with the probe head at the top of the image; dorsal is to the left and ventral to the right of the image. Centimetre dot markers are displayed on the margin of the images and should be consulted to ascertain the depth of field presented. The chest wall of adult sheep was approximately 1–1.5 cm thick. An air interface, created by aerated lung parenchyma reflects sound waves and appears as a bright white (hyperechoic) linear echo. The sonogram below the white linear echo may contain equidistant reverberation artefacts which are of no clinical significance. The area visualized below the linear echo, including the reverberation artefacts, does not represent lung parenchyma; thus the initial ultrasound machine setting with a 5 MHz sector transducer was 8 cm which examined approximately 1–1.5 cm of chest wall then pleurae and superficial lung parenchyma. The 5 MHz linear transducer used had a field depth of 7–9 cm.

**Figure 1:**
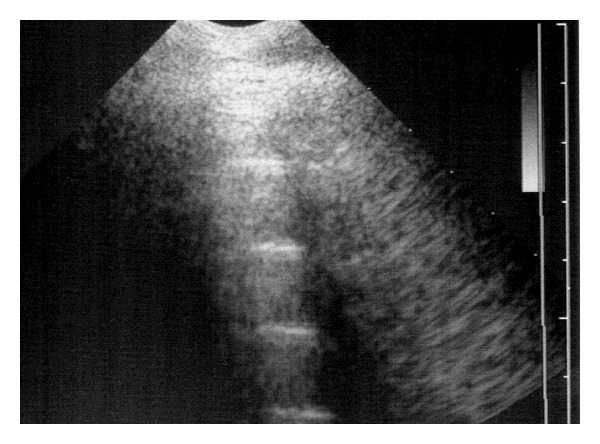
5 MHz sector scanner. The probe head is at the top of the image; dorsal is to the left. Centimetre gradations are indicated on the margin. The surface of normal aerated lung (visceral or pulmonary pleura) of normal sheep is characterized by the continuous white linear echo. Equally-spaced reverberation artefacts are often visible below the visceral pleura.

**Figure 2:**
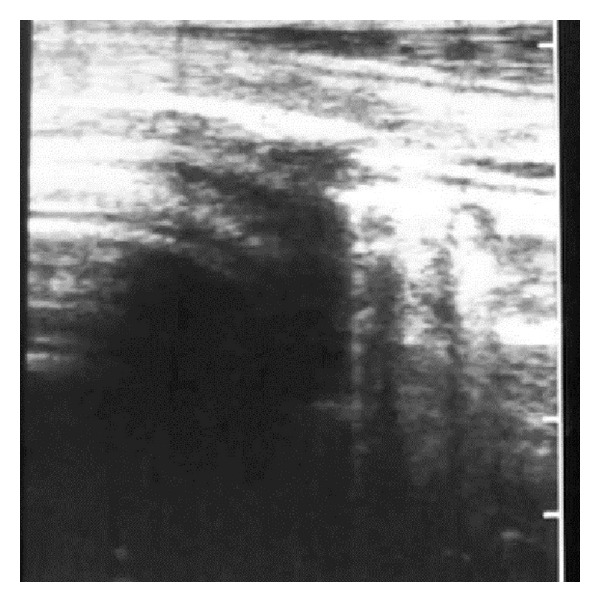
5 MHz linear scanner. The dorsal (left) half of the probe head is positioned over a rib causing shadowing; normal lung surface is apparent ventrally (right).

The surface of normal aerated lung (visceral or pulmonary pleura) was characterized by the uppermost white linear echo with equally-spaced reverberation artefacts below this line (Figure 1). Careful placement of the large linear probe head was necessary to avoid the ribs (Figure 2). In normal adult sheep (around 50–80 kg) the visceral pleura was observed moving approximately 3 mm in a vertical plane during respiration. Comet-tail artefacts represent a series of closely-spaced discrete echoes indicating the focal accumulation of a small amount of highly reflective material, often gas bubbles. The chest wall was approximately 1–1.5 cm wide.

### 3.2. Superficial Lung Abscesses

**Figure 3:**
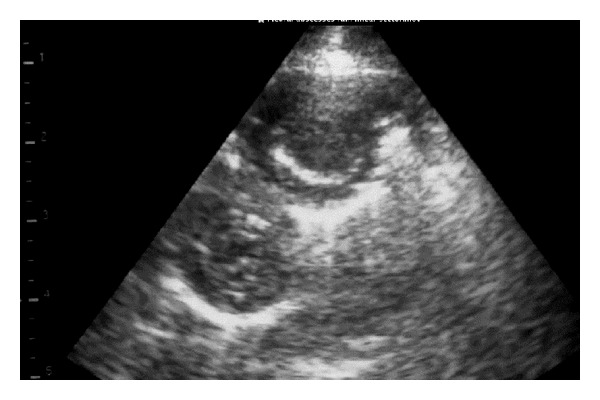
5 MHz sector scanner. 2 cm diameter pleural abscess appear as anechoic areas containing numerous hyperechoic dots bordered distally by broad hyperechoic concave lines representing the capsules.

**Figure 4:**
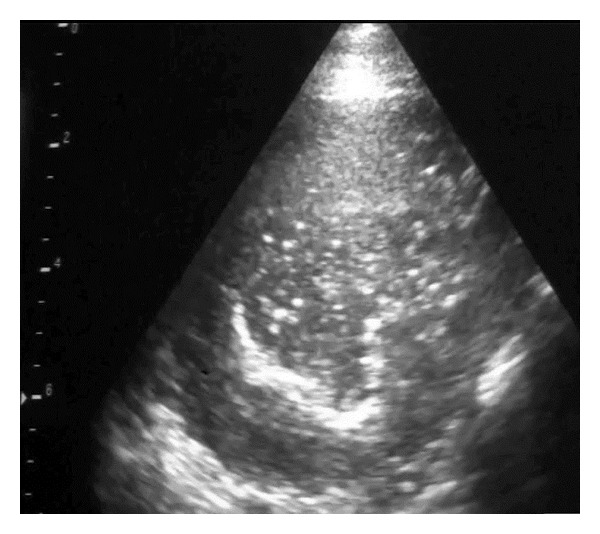
5 MHz sector scanner. Sonogram of a large lung abscess which extends 8 cm into the lung parenchyma.

The hyperechoic linear echo representing the normal visceral pleura was lost with superficial lung abscess which appeared as an uniform anechoic area containing many hyperechoic dots representing gas echoes bordered by a broad concave white abscess capsule (Figures 3 and 4). The abscess extended for 7 cm from the chest wall in Figure 4.

### 3.3. Fibrinous Pleurisy

**Figure 5:**
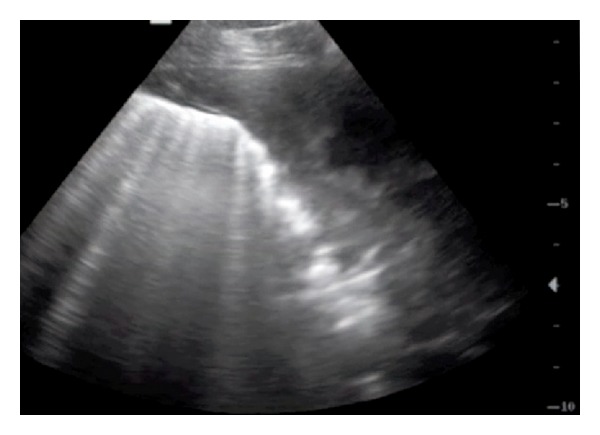
5 MHz sector scanner. The visceral pleura appears broader and more hyperechoic than normal due to acoustic enhancement by the pleural exudates with fibrin extending to 3 cm present on the parietal and visceral pleurae.

**Figure 6:**
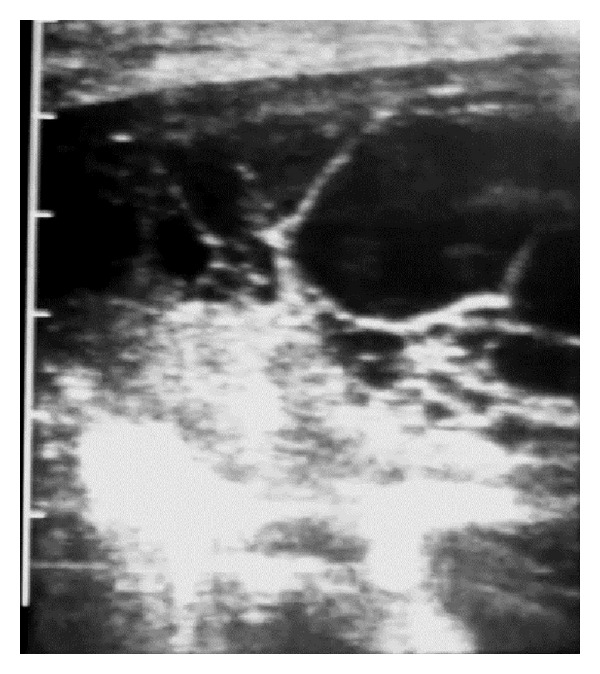
5 MHz linear scanner. The anechoic area containing a hyperechoic fibrinous matrix extends beyond the 7 cm depth range of the linear scanner.

The visceral pleura appeared broader and more hyperechoic than normal in one sheep due to acoustic enhancement by the pleural exudate (Figure 5) with fibrin extending to 3 cm present on the parietal and visceral pleurae. The visceral pleura could not be imaged in one sheep because the 7 cm wide anechoic area containing a hyperechoic fibrinous matrix extended beyond the depth range of the linear scanner (Figure 6).

### 3.4. Ovine Pulmonary Adenomatosis (OPA)

**Figure 7:**
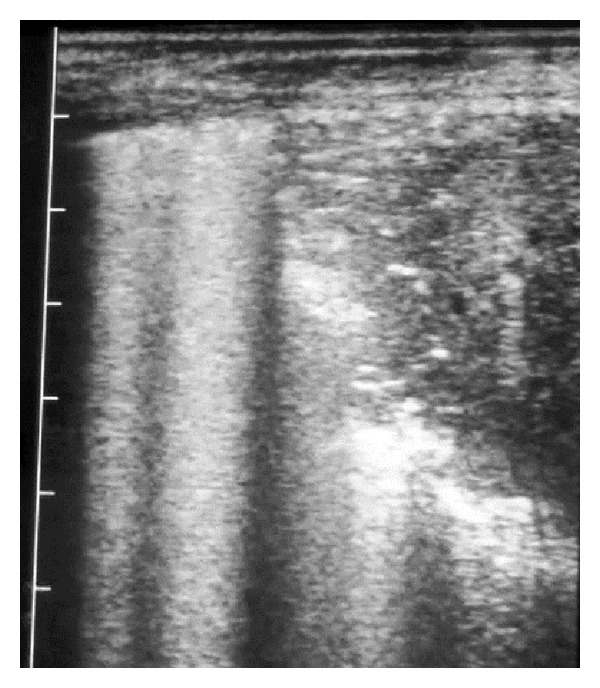
5 MHz linear scanner. Abrupt loss of the bright linear echo formed by normal aerated lung tissue (visceral or pulmonary pleura) to be replaced by a large hypoechoic area in the ventral lung.

**Figure 8:**
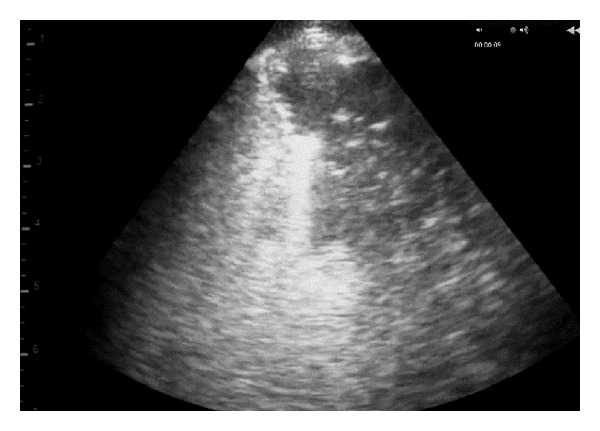
5 MHz sector scanner. Abrupt loss of the bright linear echo formed by normal aerated lung tissue (visceral or pulmonary pleura) to be replaced by a large hypoechoic area in the ventral lung.

**Figure 9:**
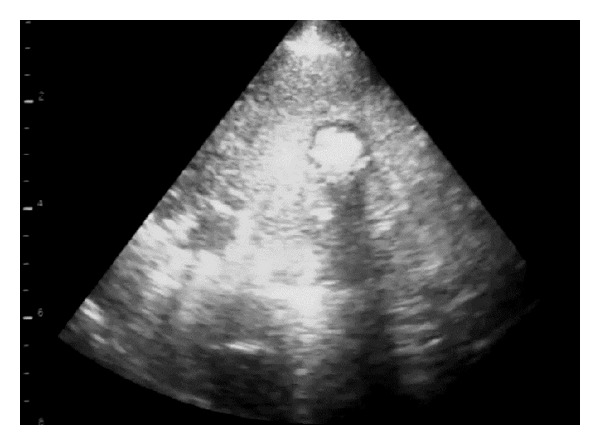
5 MHz sector scanner. 2 cm diameter abscess dorsally (to left) within the OPA tumour mass; shadowing was attributed to fibrosis/calcification of a necrotic centre within the OPA lesion.

The first indication of change in the lung parenchyma caused by OPA was the abrupt loss of the bright linear echo formed by normal aerated lung tissue (visceral or pulmonary pleura) to be replaced by a large hypoechoic area in the ventral margins of the lung lobes at the 5th or 6th intercostal spaces (Figures 7 and 8). The dorsal margin of sonographic change representing that the extent of the OPA lesion was similar for either 5 MHz linear or sector scanners. The hypoechoic areas, corresponding to lung tissue invaded by tumour cells causing consolidation (Figures 7 and 8), allowed the distribution of the OPA lesions to be accurately defined during the ultrasonographic examination. Focal hyperechoic areas, identified within the more cellularly-dense hypoechoic areas, represented large airways. Abscesses were readily identified within the tumour mass of several OPA cases; shadowing was attributed to fibrosis/calcification of necrotic centres within the tumour (Figure 9).

Consecutive daily treatment with procaine penicillin for 30 days was successful in all six sheep identified with pleural/superficial lung abscesses measuring 2–8 cm in diameter; only one of two sheep with more extensive lesions recovered.

## 4. Discussion

Chronic bacterial infection of the respiratory tract in sheep usually presents with weight loss over several weeks/months and an increased respiratory rate [11]. A wide range of descriptors has been used to describe abnormal lung sounds in sheep including increased vesicular sounds for a ram with severe chronic suppurative pleuropneumonia [12] and wheezing, rubbing vesicular, and murmuring sounds in sheep with bacterial respiratory infections, followed by absence of residual bronchial catarrh in the same sheep during recovery [13]. Authors in more recent papers [14, 15] have limited their descriptions of abnormal auscultation findings of the respiratory tract to their distribution rather than character. The present study concurs with earlier published findings [5] that auscultation does not detect any abnormal sounds in sheep with lung abscesses; marked fibrinous pleurisy caused attenuation of sounds relative to the contralateral normal lung and no sounds were heard resembling the description of pleural frictions rubs.

Ultrasonographic examination of the chest accurately defined superficial lung pathology in agreement with previous work [16, 17]. Ultrasonography was most helpful in the definitive diagnosis of superficial lung abscesses where the anechoic areas containing multiple hyperechoic dots bordered distally by a broad hyperechoic capsule were readily detected but generated no adventitious lung sounds. Daily treatment with procaine penicillin for 30 days was successful in all six sheep identified with pleural/superficial lung abscesses measuring 2–8 cm in diameter; only one of two sheep with more extensive lesions recovered. Success was defined as a rapid return to normal appetite, marked improvement in body condition, and normal respiratory rate. Penicillin is the antibiotic of choice for chronic respiratory disease in cattle and sheep because of the frequent isolation of* Arcanobacterium pyogenes* [11, 18].* Arcanobacterium pyogenes* was the most common bacterial isolate from chronic suppurative pneumonia cases in cattle and such chronic infections were treated with a 4–6-week course of procaine penicillin with reasonable success [8]. A 4–6-week duration of daily penicillin injections is necessary because of the chronicity of infection and time-dependent action of this antibiotic [11]. Other antibiotic treatments could include ceftiofur, amoxicillin, and amoxicillin/clavulanic acid combination but these regimens would prove considerably more expensive.

Lesions of ovine pulmonary adenocarcinoma (OPA) were sharply demarcated sonographically from normal lung where the hypoechoic areas extended 6–8 cm into the lung parenchyma in the cranioventral lung lobes and had the sonographic appearance of liver (hepatoid change). Such lesions cannot be accurately delineated by auscultation findings alone [5, 6]. The ultrasonographic diagnosis of OPA was confirmed at necropsy in all 21 cases in the present study; there were no false positive diagnoses. Ultrasound examination may play an important role in identifying OPA lesions and help prevent spread in the UK both for economic and animal welfare reasons.

## 5. Conclusions

Accurate identification and distribution of pleural and superficial lung pathology necessitated ultrasonographic examination; auscultation failed to identify common lesions including OPA. With some experience, systematic ultrasound examination of the ovine chest takes no more than 5 minutes. Long-term penicillin therapy was successful in 7 of 8 cases of pleural/superficial lung abscesses.
